# Supplementary material for: Bio-orthogonal Glycan Imaging of Cultured Cells and Whole Animal C. elegans with Expansion Microscopy
Source: ACS Cent Sci. 2024 Nov 23;11(2):193–207. doi: 10.1021/acscentsci.4c01061 (PMC11868961; doi:10.1021/acscentsci.4c01061)
Supplement: Supplementary file 2 — oc4c01061_si_002.pdf [file oc4c01061_si_002.pdf]

Supplemental Notes for Bio-orthogonal Glycan Imaging of Cultured Cells and Whole Animal *C. elegans* with Expansion Microscopy

Authors:

Joe Chin-Hun Kuo[1,*], Marshall J. Colville[1,*], Michelle R. Sorkin[1,*], Jacky Lok Ka Kuo[1], Ling Ting Huang[1], Dana N. Thornlow[1], Gwendolyn M. Beacham[2], Gunther Hollopeter[2], Matthew P. DeLisa[1,3,4], Christopher A. Alabi[1,#,$] and Matthew J. Paszek[1,3,5,6,#,+]

[1]Robert Frederick Smith School of Chemical and Biomolecular Engineering, Cornell University, Ithaca, NY 14853, USA

[2]Department of Molecular Medicine, Cornell University, Ithaca, NY 14853, USA

[3]Meinig School of Biomedical Engineering, Cornell University, Ithaca, NY 14853, USA

[4]Cornell Institute of Biotechnology, Cornell University, Ithaca, NY, USA

[5]Field of Biophysics, Cornell University, Ithaca, NY 14853, USA

[6]Kavli Institute at Cornell for Nanoscale Science, Ithaca, NY 14853, USA

[#]Corresponding author

*Authors contributed equally

[$]email: caa238@cornell.edu

[+]email: mjp31@cornell.edu

Expansion Microscopy Probe Synthesis Methods

Synthesis of Linker Core

Synthesis of tert-butyl N-methyl-N-(prop-2-en-1-yl)carbamate

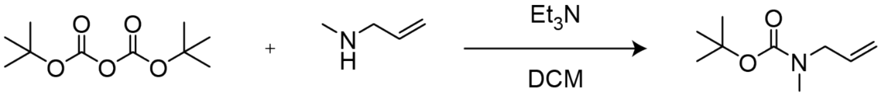

N-allylmethylamine (427 mg, 6 mmol) was dissolved at 151 mM in dichloromethane (DCM). To this solution was added 1.2 eq. of $E_{t3}N$ (729 mg, 7.2 mmol) and the mixture was allowed to equilibrate for 10 minutes on ice. Next, 1.2 eq. of di-tert-butyl dicarbonate (1090.9 mg, 5 mmol) dissolved at 1.6 M in DCM was added over 5 minutes. The final concentration of N-allylmethylamine was 132 mM. The mixture was left on ice for 1 hour and then removed from the ice and reacted at room temperature overnight. The reaction was quenched with 100 mL of water and extracted with DCM (100 mL, 3x). The DCM layer was collected and concentrated under vacuum in 82% yield. The crude product was used without further purification. The product was characterized by ^{1}H NMR and LC-MS (*m/z* calculated: 116.07 observed: 116.00 $[M\text{-t-Butyl+H}]^+$). ^{1}H NMR (400 MHz, $CDCl_3$) δ 5.82-5.70 (m, 1H), 5.18-5.07 (m, 2H), 3.87-3.74 (m, 2H), 2.86-2.76 (s, 3H), 1.52-1.37 (s, 9H).

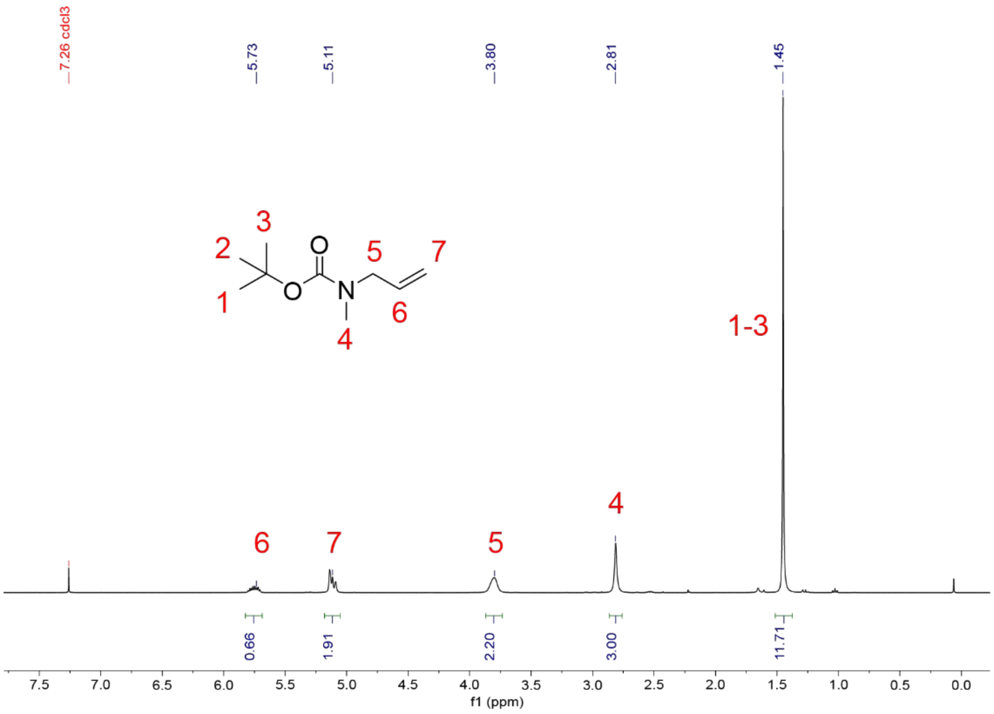

Figure 1. ^{1}H NMR (400 MHz, $CDCl_3$) of tert-butyl N-methyl-N-(prop-2-en-1-yl)carbamate.

Synthesis of S-[2-[2-(2-mercaptoethoxy)ethoxy]ethyl] ester ethanethioic acid

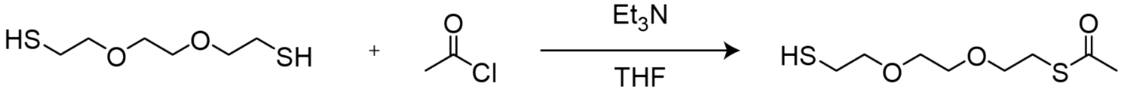

2,2'-(ethylenedioxy)diethanethiol (500 mg, 2.7 mmol) was dissolved at 150 mM in THF. To this solution was added 3 eq. of Et_3N (832 mg, 8.2 mmol) and the mixture was allowed to equilibrate on ice for 10 minutes. Next, 1.05 eq. of acetyl chloride (216 mg, 2.9 mmol) dissolved at 1.6 M in THF was added over 2 hours. The mixture was then removed from the ice and reacted at room temperature overnight. The reaction was quenched with 100 mL of water and extracted with EtOAc (100 mL, 3x). The EtOAc layer was collected and concentrated under vacuum, and the product was purified by flash chromatography (12 g silica, 0-40% ethyl acetate in hexanes) in 42% yield. The product was characterized by ^{1}H NMR. ^{1}H NMR (400 MHz, $CDCl_3$) δ 3.64-3.54 (m, 8H), 3.11-3.05 (t, 2H), 2.72-2.64 (q, 2H), 2.35-2.29 (s, 3H), 1.61-1.54 (t, 1H).

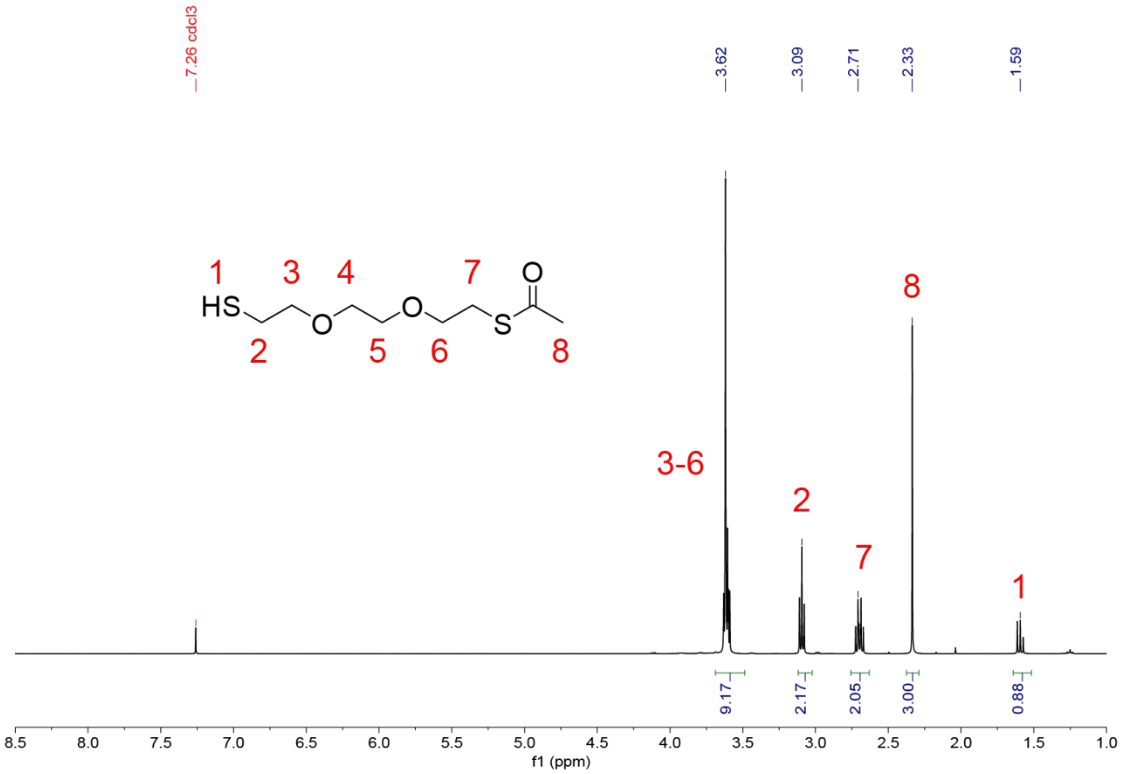

Figure 2. ^{1}H NMR (400 MHz, $CDCl_3$) of *S*-[2-[2-(2-mercaptoethoxy)ethoxy]ethyl] ester ethanethioic acid.

Synthesis of 2,[2-(2-azidoethoxy)ethoxy]ethyl-4-methylbenzenesulfonate

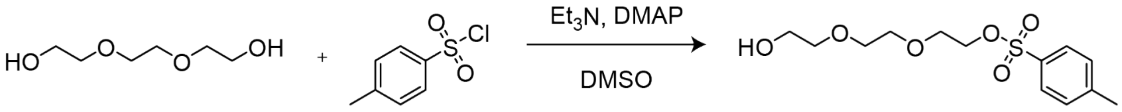

p-Toluenesulfonyl chloride (3.5 g, 18.4 mmol) was dissolved at 175 mM in dichloromethane (DCM). Separately, 4 eq. of triethylene glycol (11 g, 73 mmol) was dissolved at 110 mM in DCM. To this solution was added 1.05 eq. of Et_3N (1.95 g, 19 mmol) and 0.02 eq. of 4-dimethylaminopyridine (DMAP, 46 mg, 0.3 mmol), and the mixture was allowed to equilibrate for 10 minutes on ice. The solution of p-toluenesulfonyl chloride in DCM was then added dropwise to the mixture over 2 hours. The mixture was subsequently removed from the ice and reacted at room temperature overnight. The reaction was quenched with 100 mL of water and extracted with DCM (100 mL, 3x). The DCM layer was collected and concentrated under vacuum in 98% yield. The crude product was used without further purification. The product was characterized by ^1H NMR and LC-MS (*m/z* calculated: 305.20 observed: 305.09 $[M+H]^+$). ^1H NMR (400 MHz, $CDCl_3$) δ 7.82-7.75 (d, 2H), 7.37-7.30 (d, 2H), 4.19-4.08 (m, 2H), 3.78-3.52 (br, 10H), 2.47-2.40 (s, 3H).

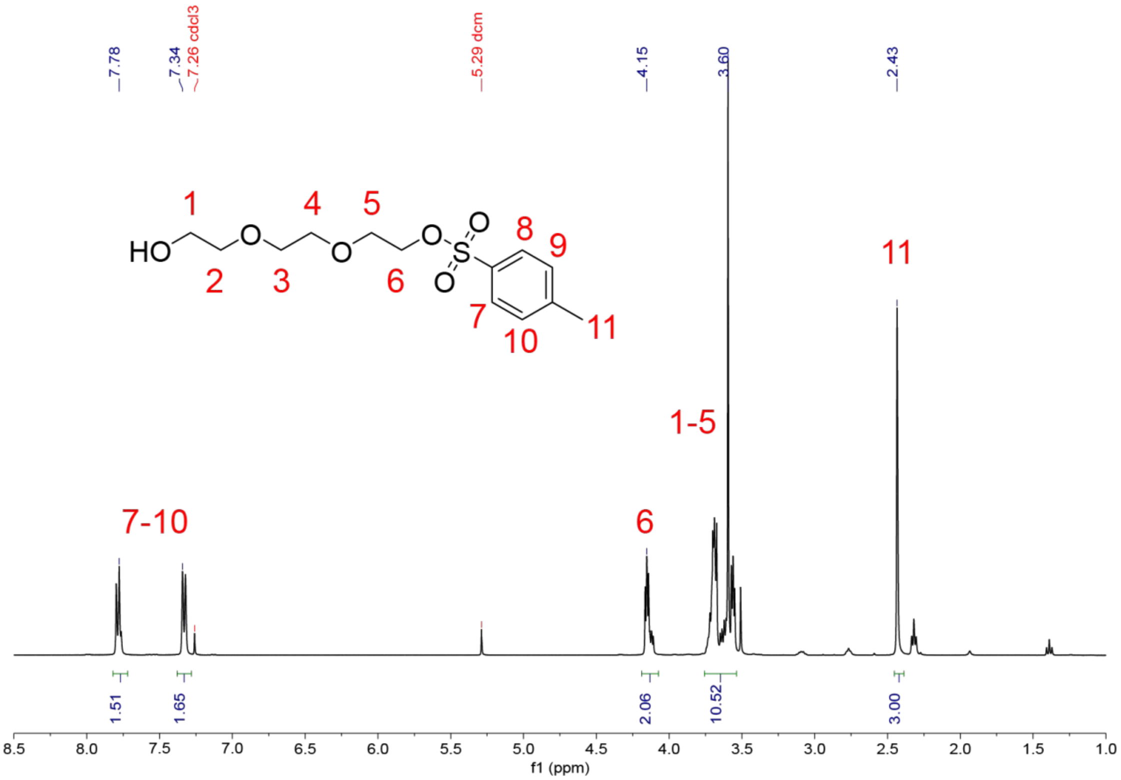

Figure 3. ^1H NMR (400 MHz, $CDCl_3$) of 2,[2-(2-azidoethoxy)ethoxy]ethyl-4-methylbenzenesulfonate.

Synthesis of 2-(2-(2-azidoethoxy)ethoxy) ethanol

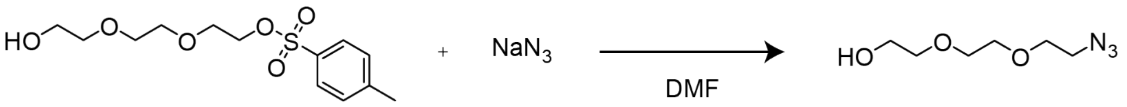

2,[2-(2-azidoethoxy)ethoxy]ethyl-4-methylbenzenesulfonate was dissolved at 739 mM in dry dimethylformamide (DMF). To this solution was added 2 eq. of sodium azide (2 g, 32 mmol) and the mixture was reacted overnight at 80 °C. The mixture was then concentrated under vacuum and the residue was resuspended in diethyl ether and filtered through celite. The ether was collected and concentrated under vacuum in 96% yield. The crude product was used without further purification. The product was characterized by ^{1}H NMR. ^{1}H NMR (400 MHz, $CDCl_3$) δ 3.74-3.70 (t, 2H), 3.69-3.64 (br, 6H), 3.62-3.58 (m, 6H), 3.41-3.35 (t, 2H).

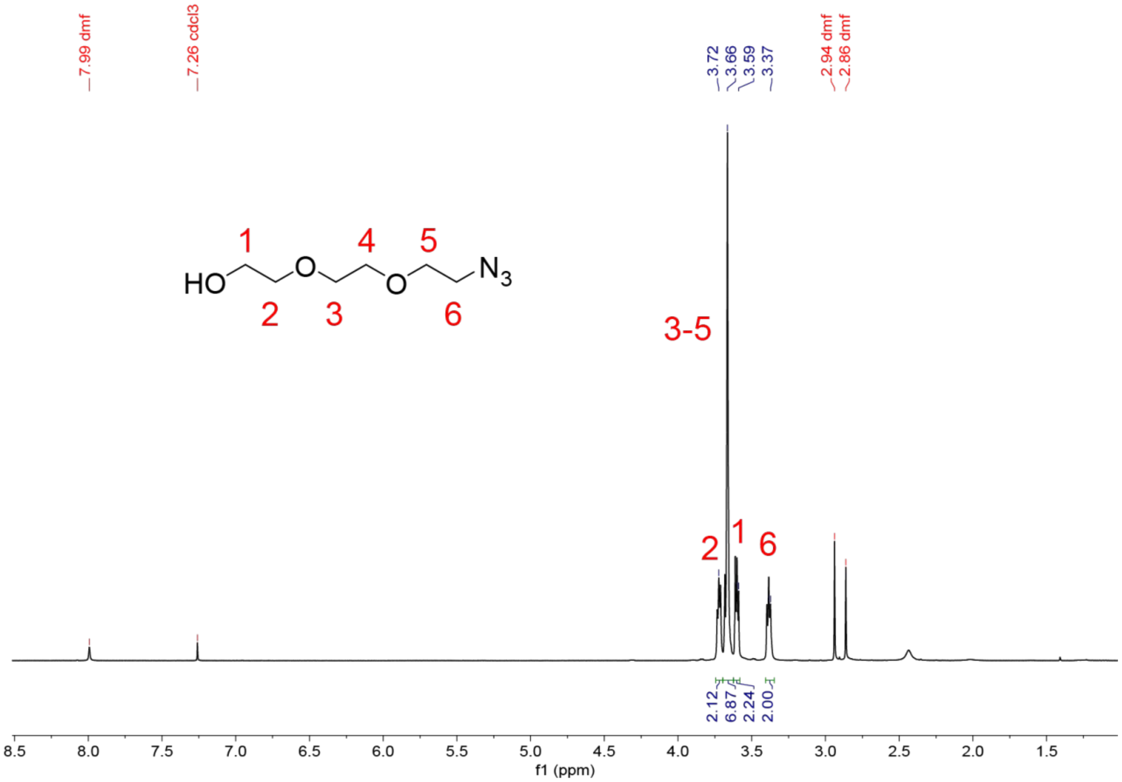

Figure 4 ^{1}H NMR (400 MHz, $CDCl_3$) of 2-(2-(2-azidoethoxy)ethoxy) ethanol.

Synthesis of 1-[2-(2-azidoethoxy)ethoxy]-2-bromoethane

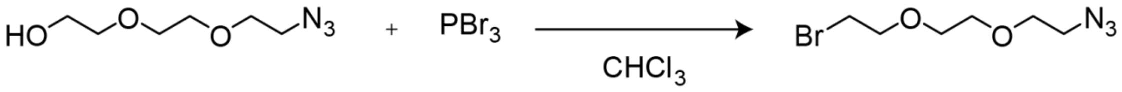

2-(2-(2-azidoethoxy)ethoxy) ethanol (1 g, 5.8 mmol) was dissolved at 342 mM in anhydrous chloroform ($CHCl_3$). To this solution was added 2 eq. of phosphorus tribromide (3.1 g, 12 mmol) over 5 minutes. The mixture was then refluxed overnight at 50 °C. The reaction was quenched on ice over 30 minutes with 75 mL of saturated sodium bicarbonate solution and extracted with $CHCl_3$ (100 mL, 3x). The $CHCl_3$ layer was collected and concentrated under vacuum in 30% yield. The crude product was used without further purification. The product was characterized by ^{13}C NMR. ^{13}C NMR (400 MHz, $CDCl_3$) δ 71.41 70.83 70.73 70.68 70.29 50.85 30.52.

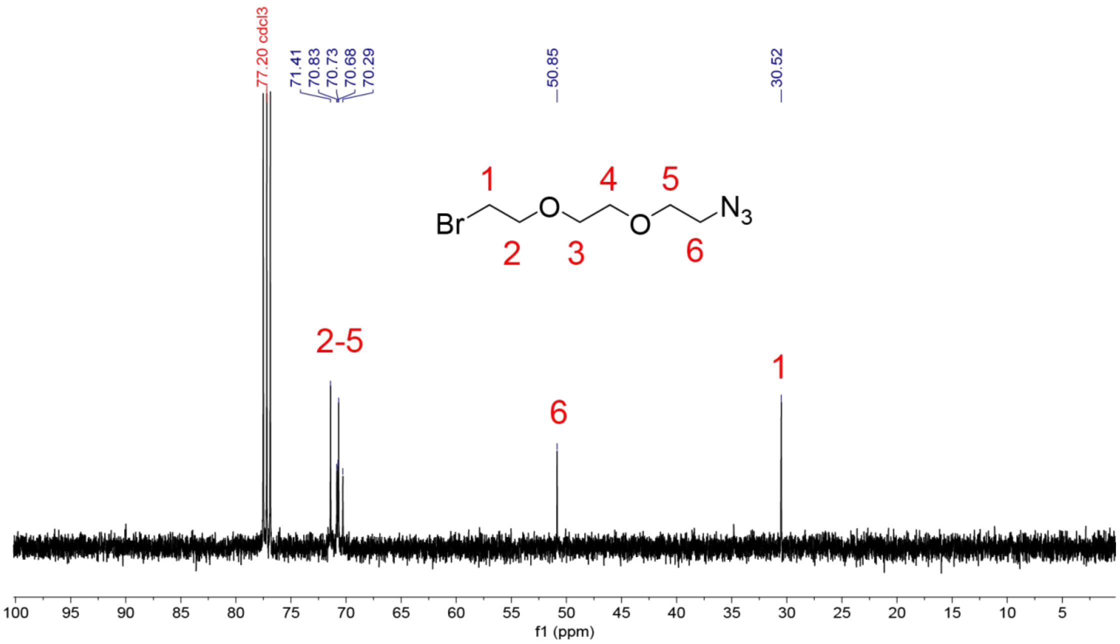

Figure 5. ^{13}C NMR (400 MHz, $CDCl_3$) of 1-[2-(2-azidoethoxy)ethoxy]-2-bromoethane.

Synthesis of N*-[2-[2-(2-azidoethoxy)ethoxy]ethyl]-2-propen-1-amine*

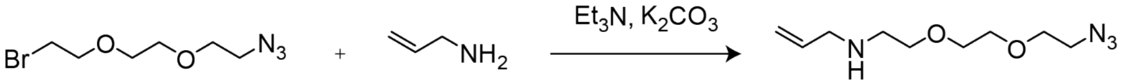

1 eq. of 1-[2-(2-azidoethoxy)ethoxy]-2-bromoethane (888 mg, 3.73 mmol) was added to 1.2 eq. of potassium carbonate (619 mg, 4.48 mmol) and 10 eq. allylamine (2.1 g, 37.3 mmol). The mixture was allowed to react overnight at room temperature. The reaction was then filtered through celite and concentrated under vacuum in 85% recovery. The crude product was used

without further purification. The product was characterized by ^{1}H NMR. ^{1}H NMR (400 MHz, $CDCl_3$) δ 5.99-5.86 (m,1H), 5.24-5.07 (dd, 1H), 3.75-3.58 (br, 8H), 3.46-3.38 (t, 2H), 3.31-3.25 (d, 2H), 2.83-2.79 (t, 2H).

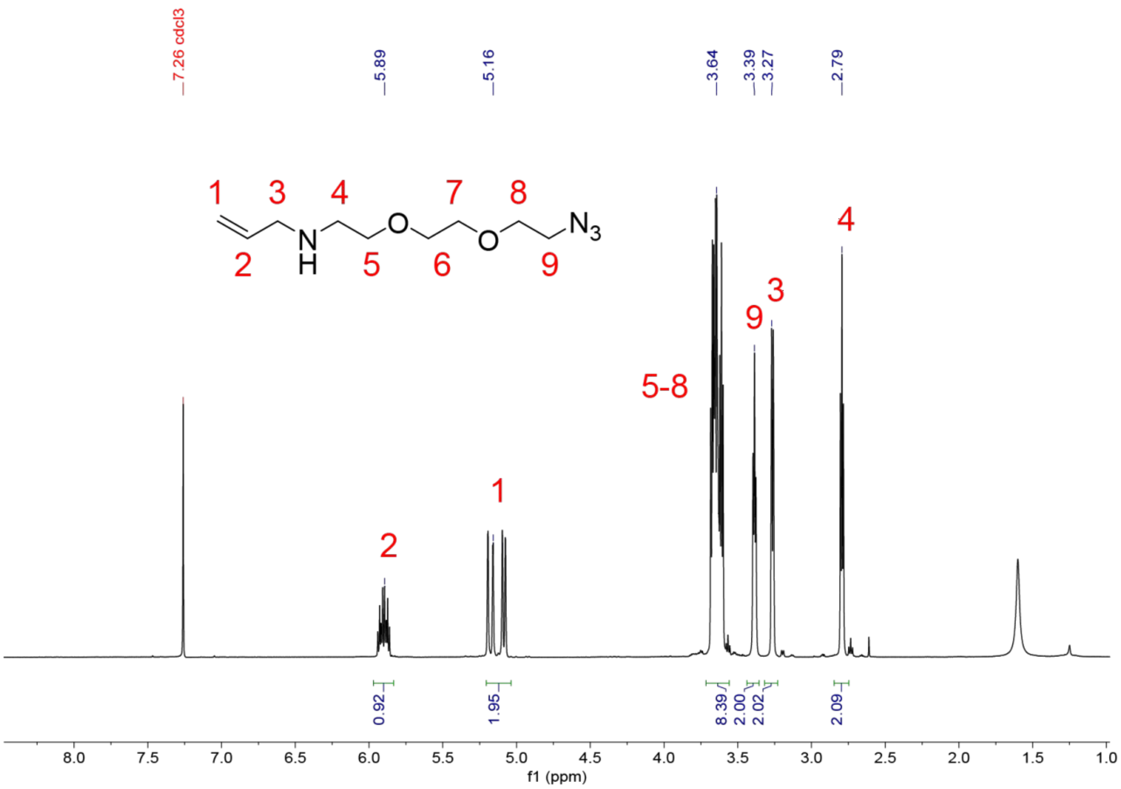

Figure 6. ^{1}H NMR (400 MHz, $CDCl_3$) of *N*-[2-[2-(2-azidoethoxy)ethoxy]ethyl]-2-propen-1-amine.

Synthesis of Compound ***(1)***

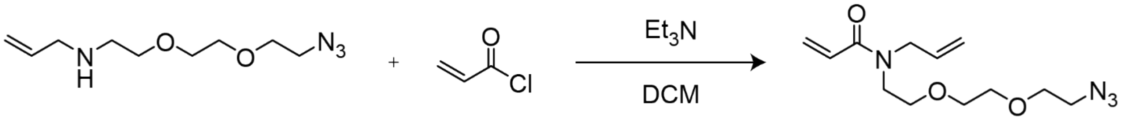

(1)

1 eq. of *N*-[2-[2-(2-azidoethoxy)ethoxy]ethyl]-2-propen-1-amine (668 mg, 3.1 mmol) was dissolved at 151 mM in dichloromethane (DCM). To the solution was added 1.1 eq. of Et_3N (347 mg, 3.4 mmol), and the mixture was allowed to equilibrate for 10 minutes on ice. Next, 1.3 eq. acryloyl chloride (367 mg, 4.1 mmol) dissolved at 1.66 M in DCM was added dropwise for 1 hour. The final concentration of *N*-[2-[2-(2-azidoethoxy)ethoxy]ethyl]-2-propen-1-amine was 132 mM. The mixture was then removed from the ice and reacted at room temperature for 1 hour. The reaction was quenched with 6 mL of water and extracted with DCM (80 mL, 3x). The DCM layer

was collected and concentrated under vacuum. The product was purified by flash chromatography (12 g silica, 0-5% MeOH in DCM) in 30% yield. The product was characterized by ^{1}H NMR and LC-MS (*m/z* calculated: 269.16 observed: 269.20 $[M+H]^+$). ^{1}H NMR (400 MHz, $CDCl_3$) δ 6.72-6.28 (m, 2H), 5.85-5.72 (m, 1H), 5.70-5.60 (m, 1H), 5.22-5.08 (m, 2H), 4.10-4.04 (m, 2H), 3.70-3.45 (m, 10H), 3.40-3.31, (t, J = 5.1 Hz, 2H).

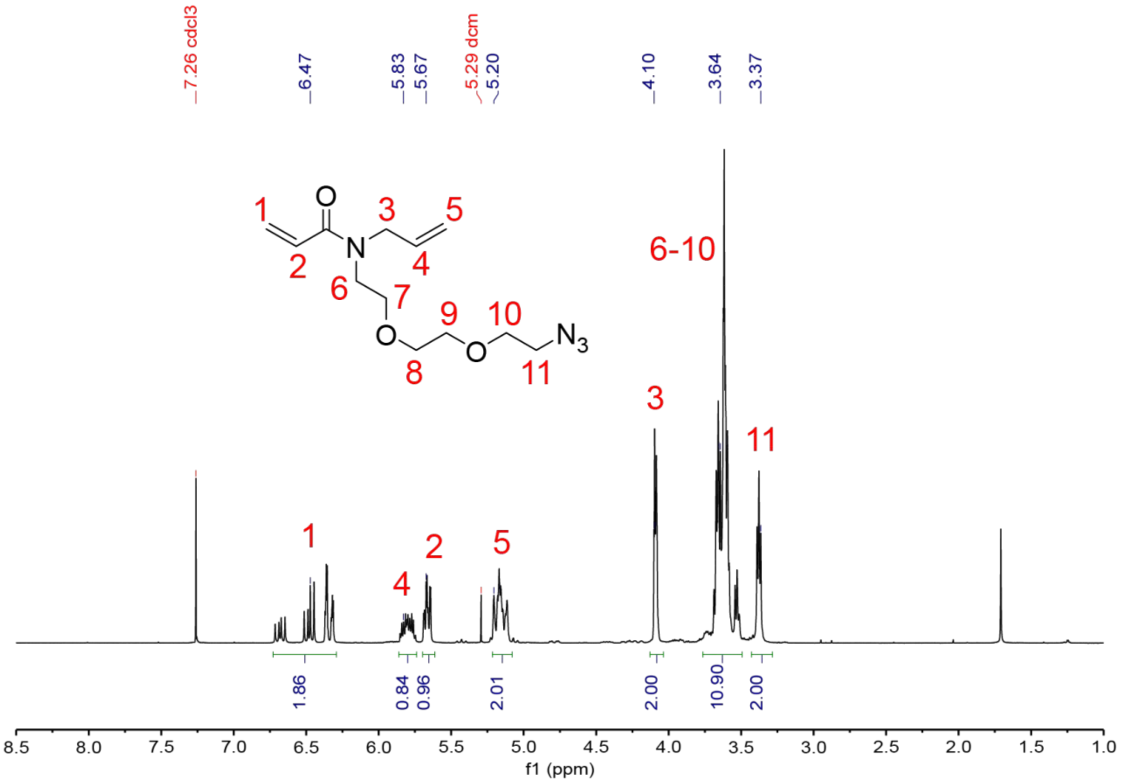

Figure 7. ^{1}H NMR (400 MHz, $CDCl_3$) of Compound **(1)**.

*Synthesis of Compound **(2)***

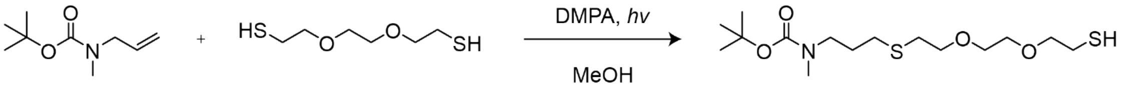

(2)

Tert-butyl N-methyl-N-(prop-2-en-1-yl)carbamate was dissolved at 500 mg/ml in methanol (MeOH). To this solution was added 0.1 eq. of 2,2-dimethoxy-2-phenylacetophenone (DMPA) and 5 eq. of 2,2'-(ethylenedioxy)diethanethiol. The final concentration of tert-butyl N-methyl-N-(prop-2-en-1-yl)carbamate was 306 mM in MeOH. The mixture was subjected to UV irradiation at 5 mW/cm^2 for 270 s. The methanol was then evaporated under reduced pressure, and the product

was purified by flash chromatography (40 g silica, 0-20% ethyl acetate in hexanes). The product was characterized by ^{1}H NMR. ^{1}H NMR (400 MHz, $CDCl_3$) δ 3.67-3.54 (m, 8H), 3.31-3.22 (t, 2H), 2.85-2.79 (s, 3H), 2.74-2.65 (m, 4H), 2.56-2.48 (t, 2H), 1.83-1.71 (q, 2H), 1.62-1.54 (t, 1H), 1.47-1.37 (s, 9H).

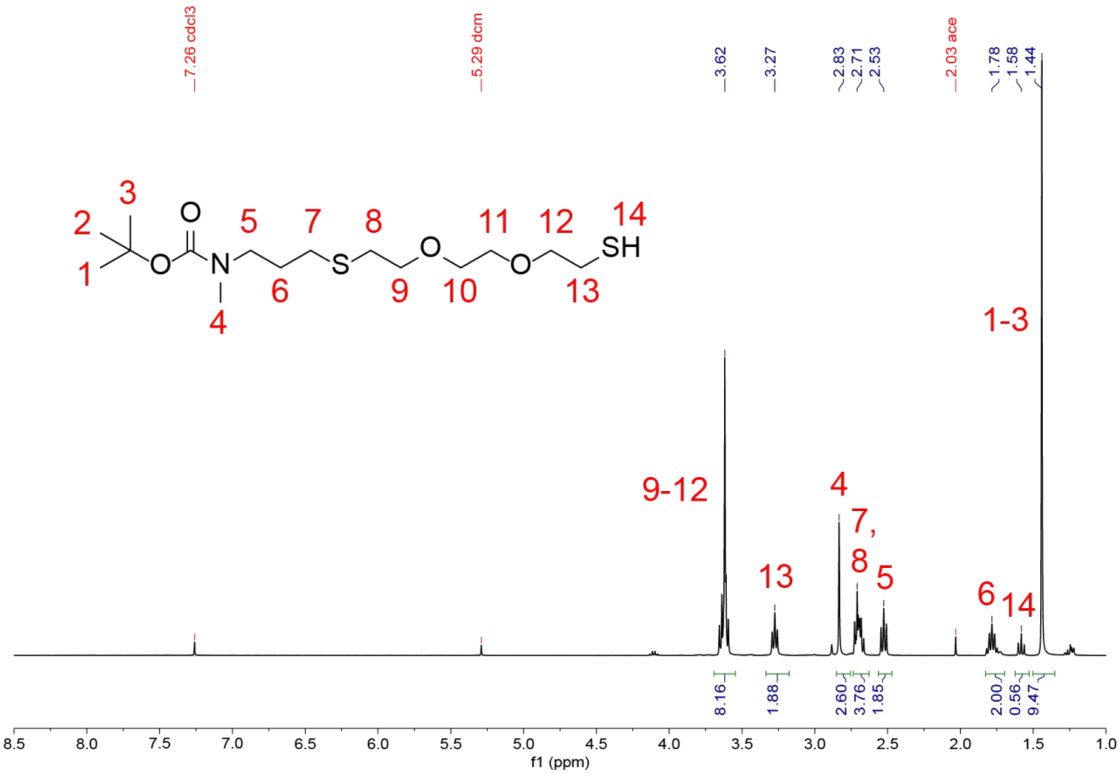

Figure 8. ^{1}H NMR (400 MHz, $CDCl_3$) of Compound **(2)**.

*Synthesis of Compound **(3)***

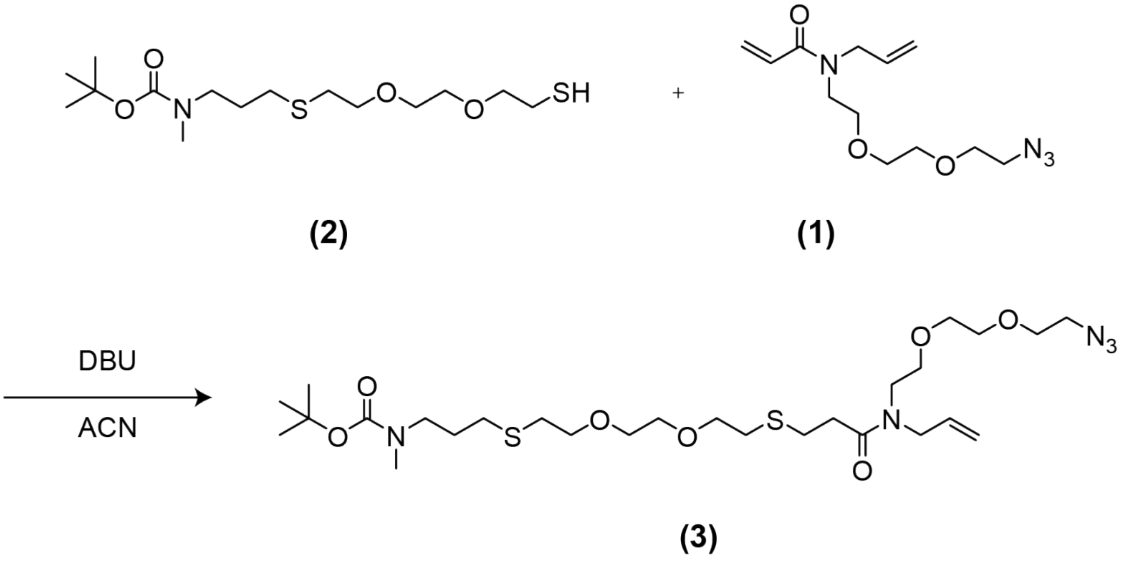

1 eq. of Compound **(2)** was mixed with 1 eq. of Compound **(1)** and 0.05 eq. 1,8-diazabicyclo[5.4.0]undec-7-ene (DBU) at a final concentration of 600 mM in acetonitrile (ACN). The mixture was reacted overnight at room temperature. The product was characterized by LC-MS (*m/z* calculated: 644.31 observed: 644.20 $[M+Na]^+$) and used without further purification.

*Synthesis of Compound **(4)***

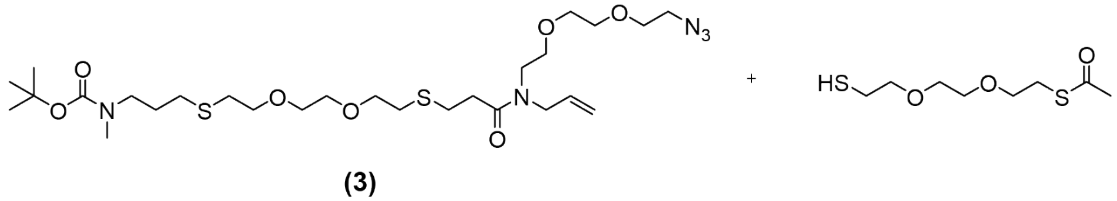

(3)

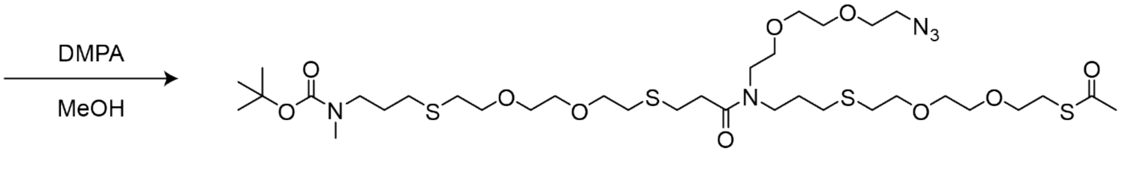

(4)

1 eq. of Compound **(3)** was mixed with 2.2 eq. of *S*-[2-[2-(2-mercaptoethoxy)ethoxy]ethyl] ester ethanethioic acid and 0.2 eq. of DMPA at a final concentration of 600 mM in MeOH. The mixture was subjected to UV irradiation at 5 mW/cm^2 for 270 s. The mixture was purified via semi-preparative RP-HPLC (mobile phase without TFA). The product eluted at 37.9 minutes and was characterized by ^1H NMR and LC-MS (*m/z* calculated: 868.37 observed: 868.20 $[M+Na]^+$). ^1H NMR (400 MHz, $CDCl_3$) δ 3.67-3.54 (br, 24H), 3.53-3.40 (m, 4H), 3.39-3.35 (t, 2H), 3.28-3.23 (m, 2H), 3.09-3.05 (t, 2H), 2.86-2.47 (m, 16H), 2.33-2.39 (s, 2H), 1.87-1.73 (m, 4H), 1.45-1.40 (s, 9H).

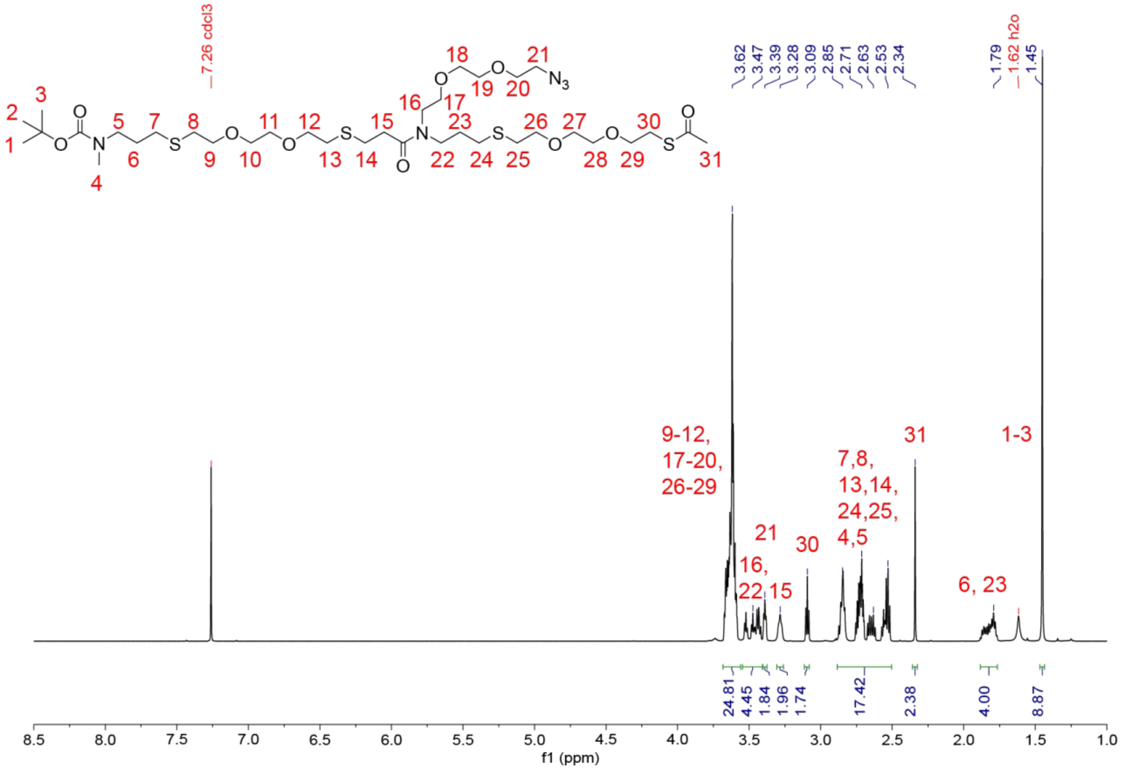

Figure 9. ^{1}H NMR (400 MHz, $CDCl_3$) of Compound **(4)**.

*Tert-butoxy carbamate (Boc) Deprotection of Compound **(4)***

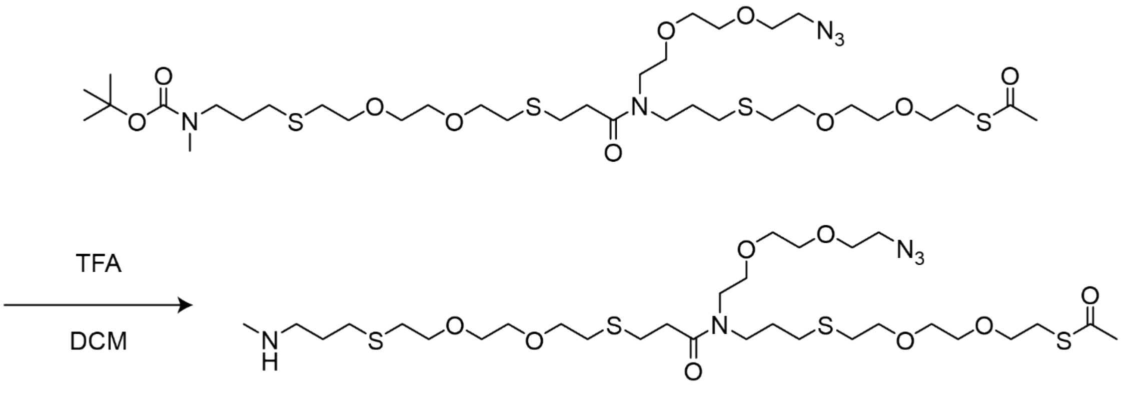

(5)

Compound **(4)** was mixed at 5 mM in 50% trifluoroacetic acid (TFA) in DCM for 1 hour. The TFA and DCM were then removed under vacuum. The product was characterized by LC-MS (*m/z* calculated: 746.33 observed: 746.20 $[M+H]^+$).

Linker Core Conjugation to Reporter, “Click” Handle, and Anchoring Group

Synthesis of 2-(phenyldithio)-ethanamine

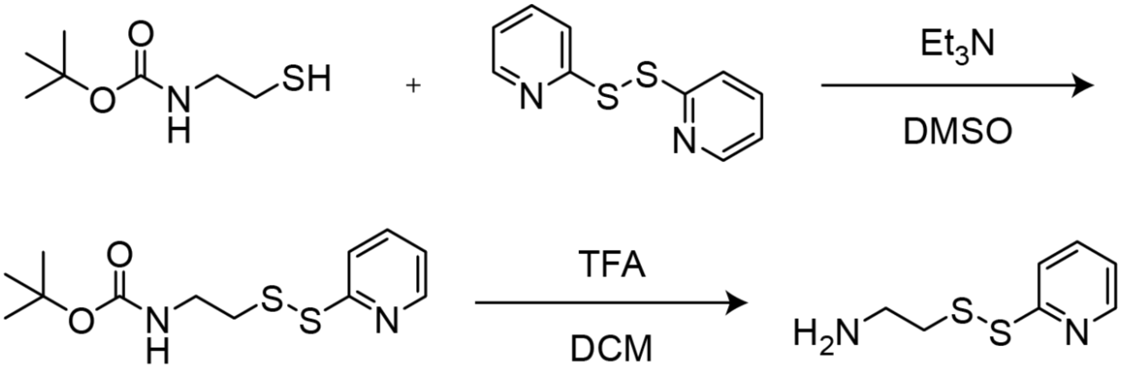

1 eq. of tert-butyl N-(2-mercaptoethyl)carbamate was mixed with 1 eq. of 2,2'-dithiodipyridine and 2 eq. of Et_3N. The final concentration of tert-butyl N-(2-mercaptoethyl)carbamate was 500 mM in DMSO. The mixture was reacted overnight at room temperature and purified via semi-preparative RP-HPLC (mobile phase with TFA). The HPLC-purified product was then mixed at 50 mM in 50% trifluoroacetic acid (TFA) in DCM for 1 hour. The TFA and DCM were then removed under vacuum to yield the desired product, 2-(phenyldithio)-ethanamine.

*Synthesis of Compound **(5)***

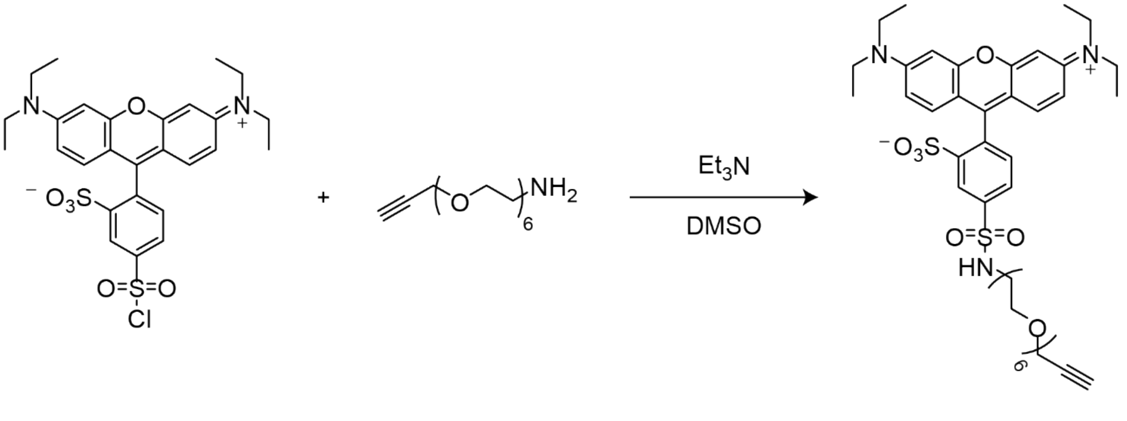

(6)

1 eq. of lissamine rhodamine B sulfonyl chloride was mixed with 2 eq. of 3,6,9,12,15,18-hexaoxahenicos-20yn-1-amine and 5 eq. of Et_3N at a final concentration of 100 mM in DMF. The mixture was reacted overnight at room temperature and purified via semi-preparative RP-HPLC. The product was characterized by LC-MS (*m/z* calculated: 860.35 observed: 860.20 $[M+H]^+$).

Synthesis of Compound ***(6)***

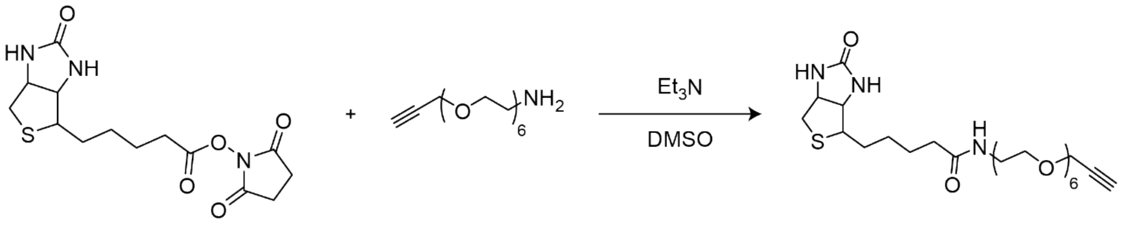

(7)

1 eq. of D-biotin *N*-succinimidyl ester was mixed with 2 eq. of 3,6,9,12,15,18-hexaoxahenicos-20yn-1-amine and 5 eq. of Et_3N at a final concentration of 100 mM in DMF. The mixture was reacted overnight at room temperature and purified via semi-preparative RP-HPLC (mobile phase with TFA). The product was characterized by LC-MS (*m/z* calculated: 546.29 observed: 546.20 $[M+H]^+$).

Synthesis of Compound ***(7)***

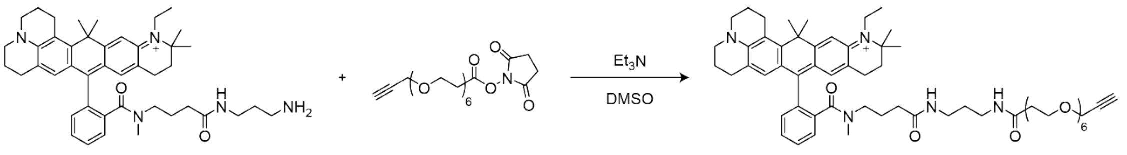

(8)

1 eq. of Atto 647N amine was mixed with 2 eq. of 2,5-dioxopyrrolidin-1-yl 4,7,10,13,16,19-hexaoxadocos-21-ynoate and 5 eq. of Et_3N at a final concentration of 100 mM in DMF. The mixture was reacted overnight at room temperature and purified via semi-preparative RP-HPLC (mobile phase with TFA). The product was characterized by LC-MS (*m/z* calculated: 1018.63 observed: 1018.40 $[M]^+$).

Synthesis of Compounds ***(9)****,* ***(10)****, and* ***(11)***

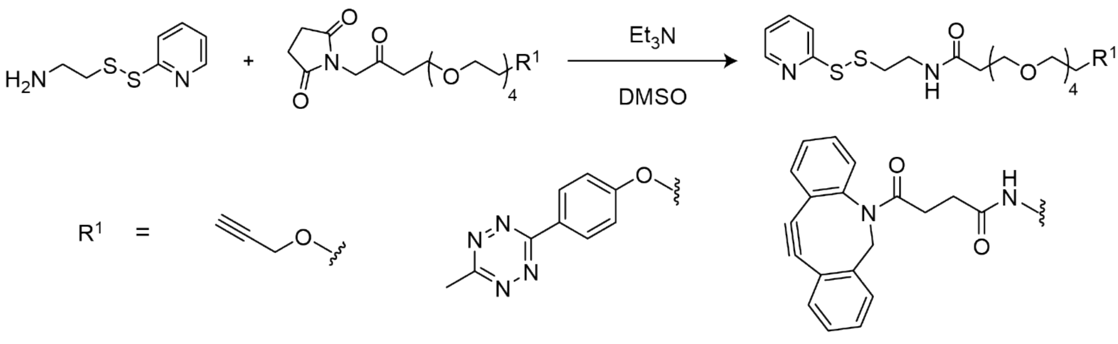

(9) **(10)** **(11)**

1 eq. of 4,7,10,13,16-pentaoxanonadec-18-ynoic acid *N*-succinimidyl ester (Alkyne-PEG4-NHS), 3-[2-[2-[2-[2-[4-(6-methyl-1,2,4,5-tetrazin-3-yl)phenoxy]ethoxy]ethoxy]ethoxy]ethoxy]-, 2,5-dioxo-1-pyrrolidinyl ester (Methyltetrazine-PEG4-NHS), or 2,5-dioxo-1-pyrrolidinyl 20-(11,12-didehydrodibenz[b,f]azocin-5(6H)-yl)-17,20-dioxo-4,7,10,13-tetraoxa-16-azaeicosanoate (DBCO-PEG4-NHS) (50 mg/ml in DMSO) was mixed with 2 eq. of 2-(phenyldithio)-ethanamine (100 mg/ml in DMSO) and 3 eq. of Et_3N at a final concentration of 80 mM in DMSO. The mixture was reacted overnight at room temperature and purified via semi-preparative RP-HPLC. The product was characterized by LC-MS (Compound **(9)** *m/z* calculated: 473.18 observed: 473.10 $[M+H]^+$; Compound **(10)** *m/z* calculated: 721.28 observed: 721.20 $[M+H]^+$; Compound **(11)** *m/z* calculated: 605.10 observed: 605.22 $[M+H]^+$).

*Synthesis of Compounds **(12)**, **(13)**, and **(14)***

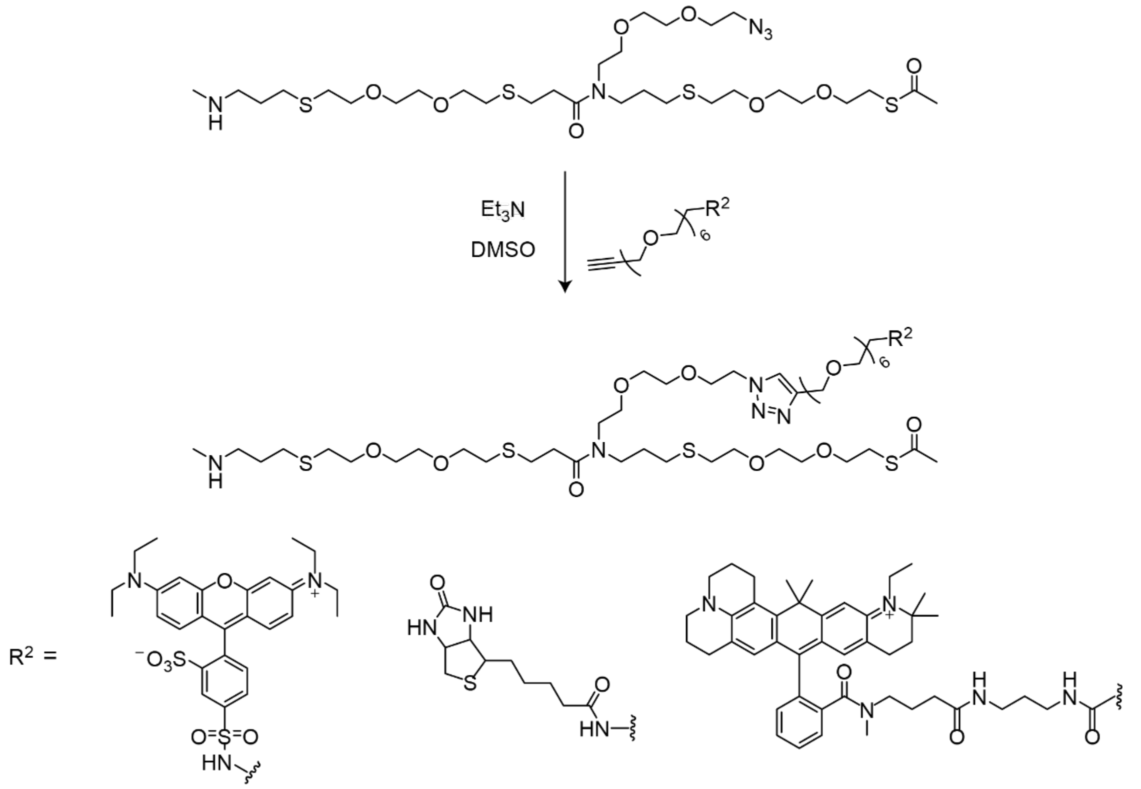

(12) **(13)** **(14)**

1 eq. of Compound **(5)** was dissolved at 15 mg/ml in DMSO. To this solution was added 1 eq. of Compound **(6)**, **(7)**, or **(8)** (160 mg/ml in DMSO), 0.25 eq. of copper sulfate (10 mg/ml in water), 0.5 eq. of tris[(1-benzyl-1*H*-1,2,3-triazol-4-yl)methyl]amine (TBTA) (50 mg/ml in DMSO), and 2.5 eq. of sodium ascorbate (30 mg/ml in water). The final concentration of Compound **(5)** was 8.5 mM in 20% water in DMSO. The mixture was reacted overnight at room temperature and purified

via semi-preparative RP-HPLC (mobile phase with TFA). The product was characterized by LC-MS (Compound **(12)** *m/z* calculated: 782.20 observed: 782.33 $[M+2H]^{2+}$; Compound **(13)** *m/z* calculated: 646.30 observed: 646.30 $[M+2H]^{2+}$; Compound **(14)** *m/z* calculated: 882.98 observed: 882.40 $[M+2H]^{2+}$).

*Conjugation of Compounds **(12)**, **(13)**, or **(14)** to Compound **(9)**, **(10)**, or **(11)** [Synthesis of **"Click" Handle-modified Oligomers**]*

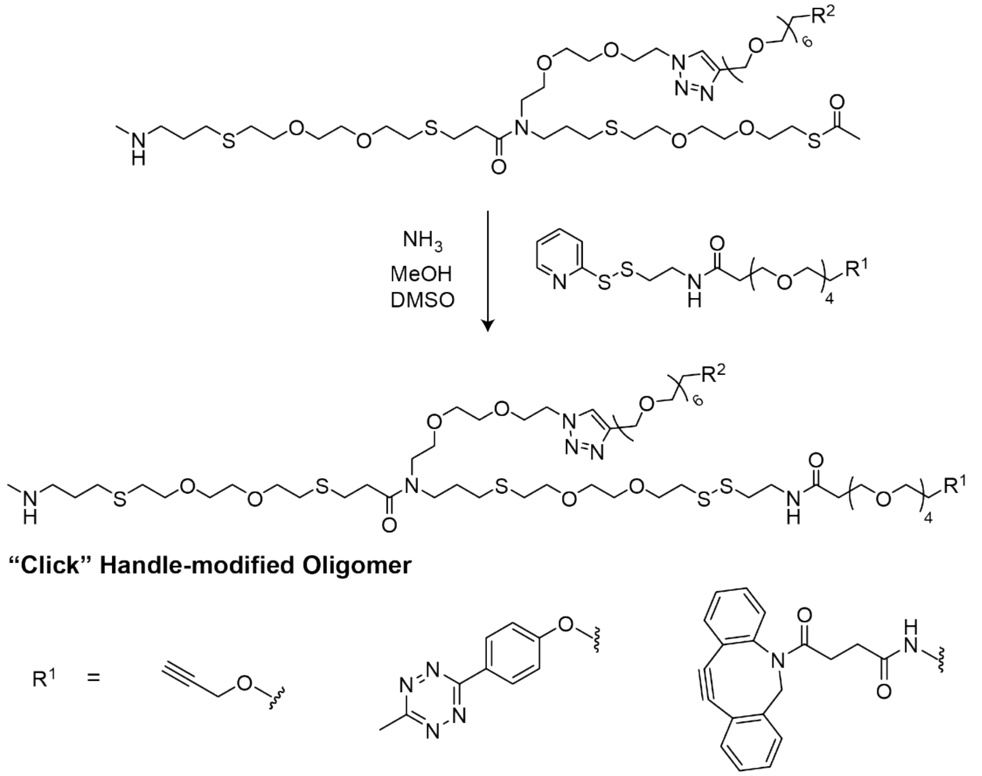

(9) **(10)** **(11)**

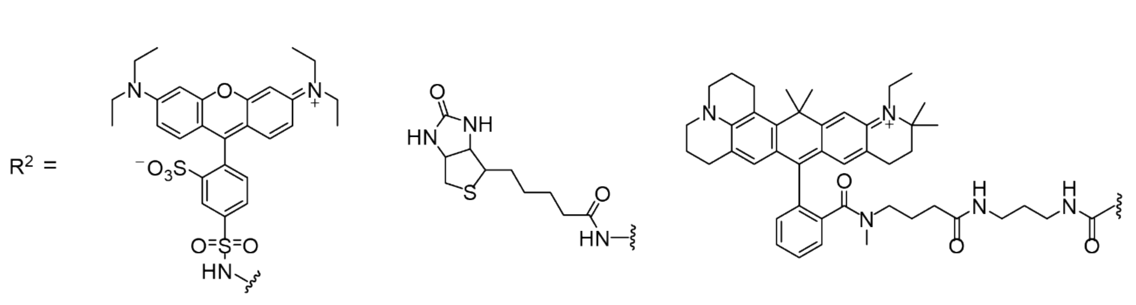

(12) **(13)** **(14)**

To 1 eq. of Compound **(12)**, **(13)**, or **(14)** was added 200 eq. of ammonia (7N in MeOH) to give a final concentration of 500 mM in MeOH. The mixture was reacted for 30 minutes at room

temperature. Next, 1.2 eq. of Compound **(9)**, **(10)**, or **(11)** was added and reacted for 2 hours at room temperature. The reaction was then purified via semi-preparative RP-HPLC (mobile phase with TFA). The product was characterized by LC-MS (Compound **(12-9)** *m/z* calculated: 962.91 observed 963.26 $[M+2H]^{2+}$; Compound **(12-10)** *m/z* calculated: 1028.93 observed: 1029.73 $[M+2H]^{2+}$; Compound **(12-11)** *m/z* calculated: 1086.95 observed 1087.30 $[M+2H]^{2+}$; Compound **(13-9)** calculated: 805.88 observed: 805.99 $[M+2H]^{2+}$; Compound **(14-9)** *m/z* calculated: 1042.44 observed: 1042.43 $[M+2H]^{2+}$).

*Conjugation of fmoc-N-amido-dPEG$_{12}$-TFP ester to "Click" Handle-modified Oligomer [Synthesis of **PEG12-Modified Oligomer**]*

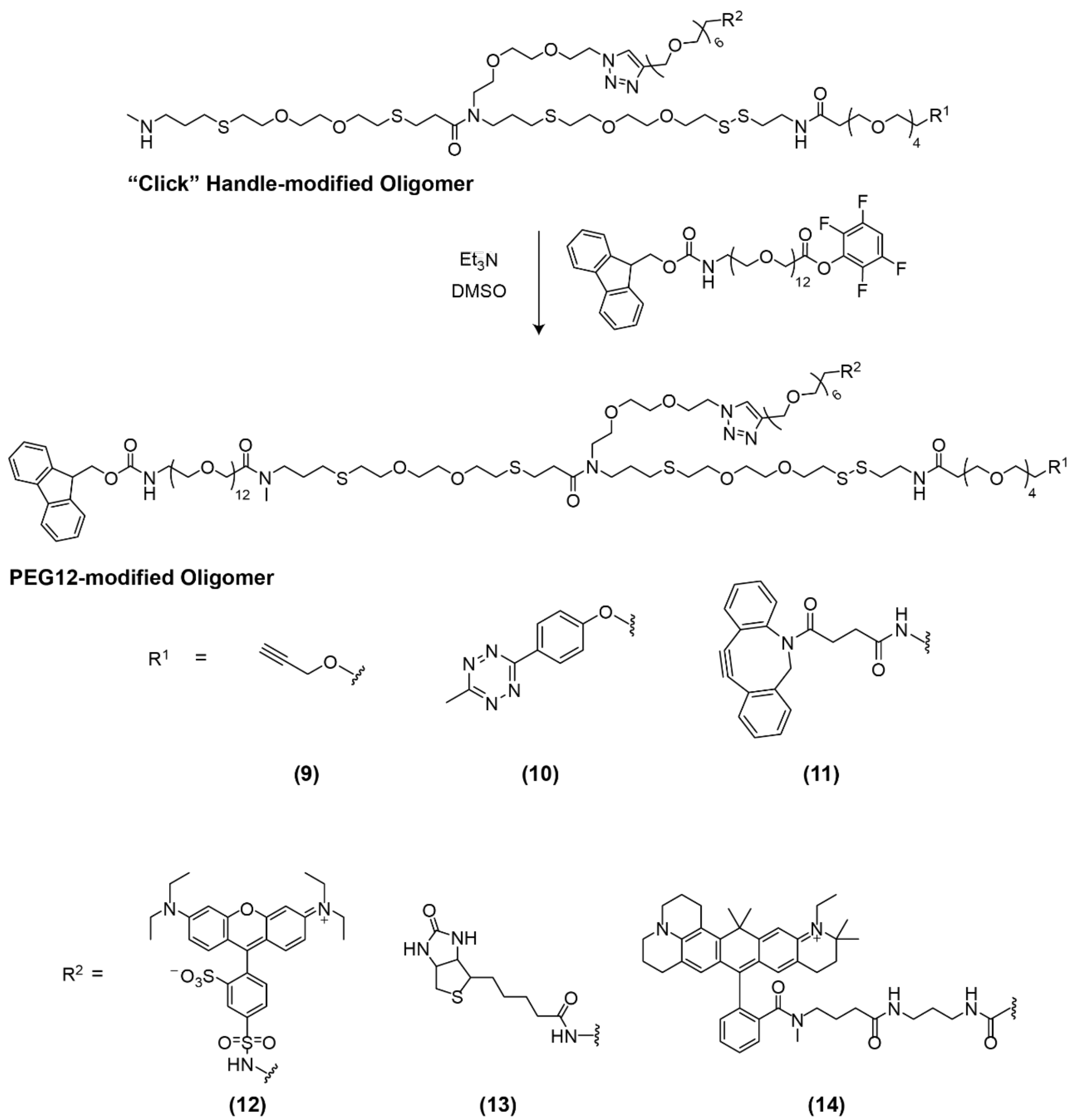

1 eq. of “Click” Handle-modified Oligomer was dissolved at 15 mg/ml in DMSO. To this solution was added 2.2 eq. of fmoc-N-amido-dPEG$_{12}$-TFP ester and 5 eq. of Et_3N. The final concentration of “Click” Handle-modified Oligomer was 5 mM in DMSO. The mixture was reacted for 1 hour at room temperature and then purified via semi-preparative RP-HPLC (mobile phase with TFA). The product was characterized by LC-MS (Compound **(PEG12-12-9)** *m/z* calculated: 1373.62 observed: 1374.31 $[M+2H]^{2+}$; Compound **(PEG12-12-10)** *m/*z calculated: 1086.95 observed: 1087.34 $[M+2H]^{2+}$; Compound **(PEG-12-12-11)** *m/z* calculated: 1439.64 observed 1440.60 $[M+2H]^{2+}$; Compound **(PEG12-13-9)** calculated: 1216.59 observed: 1217.17 $[M+2H]^{2+}$; Compound **(PEG12-14-9)** *m/z* calculated: 1453.26 observed: 1453.50 $[M+2H]^{2+}$).

*Conjugation of Methacrylic Acid NHS Ester to PEG12-modified Oligomer (Synthesis of **"Click" ExM Scaffolds**)*

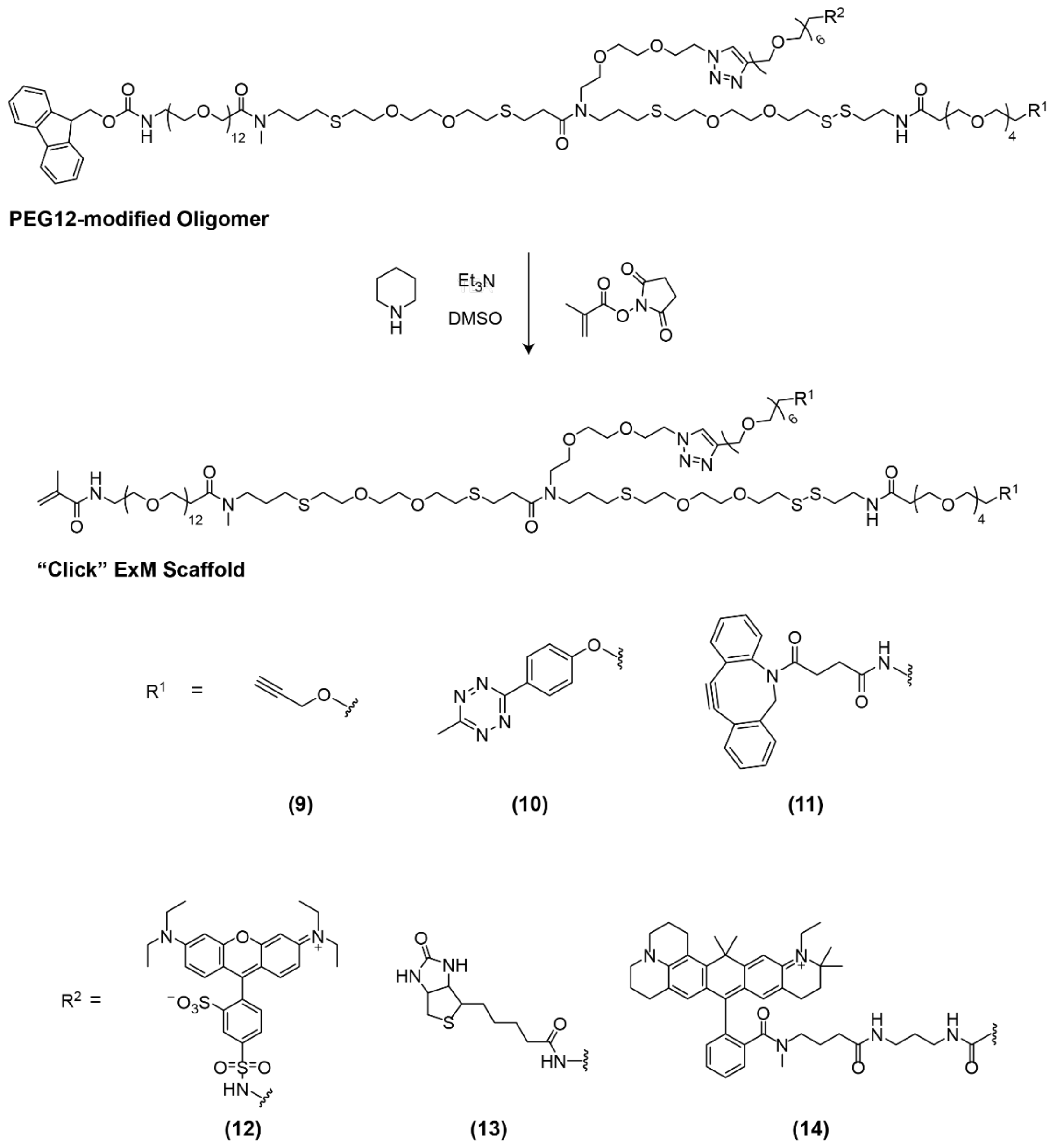

1 eq. of PEG12-modified oligomer was dissolved at 30 mg/ml in DMSO. To this solution was added 5 eq. of piperidine to give a final concentration of 69 mM in DMSO. After reacting for 1 hour at room temperature, 10 eq. of methacrylic acid NHS ester (100 mg/ml) and 15 eq. of Et_3N were added. The final concentration was 3 mM in DMSO. The mixture was reacted for 2 hours at room temperature and then purified via semi-preparative RP-HPLC (mobile phase with TFA). The product was characterized by LC-MS (Compound **(Methac-PEG12-12-9)** *m/z* calculated: 1296.60 observed: 1297.00 $[M+2H]^{2+}$; Compound **(Methac-PEG12-12-10)** *m/z* calculated: 1362.62

observed: 1363.20 $[M+2H]^{2+}$; Compound **(Methac-PEG12-12-11)** calculated: 1420.64 observed: 1421.10 $[M+2H]^{2+}$.

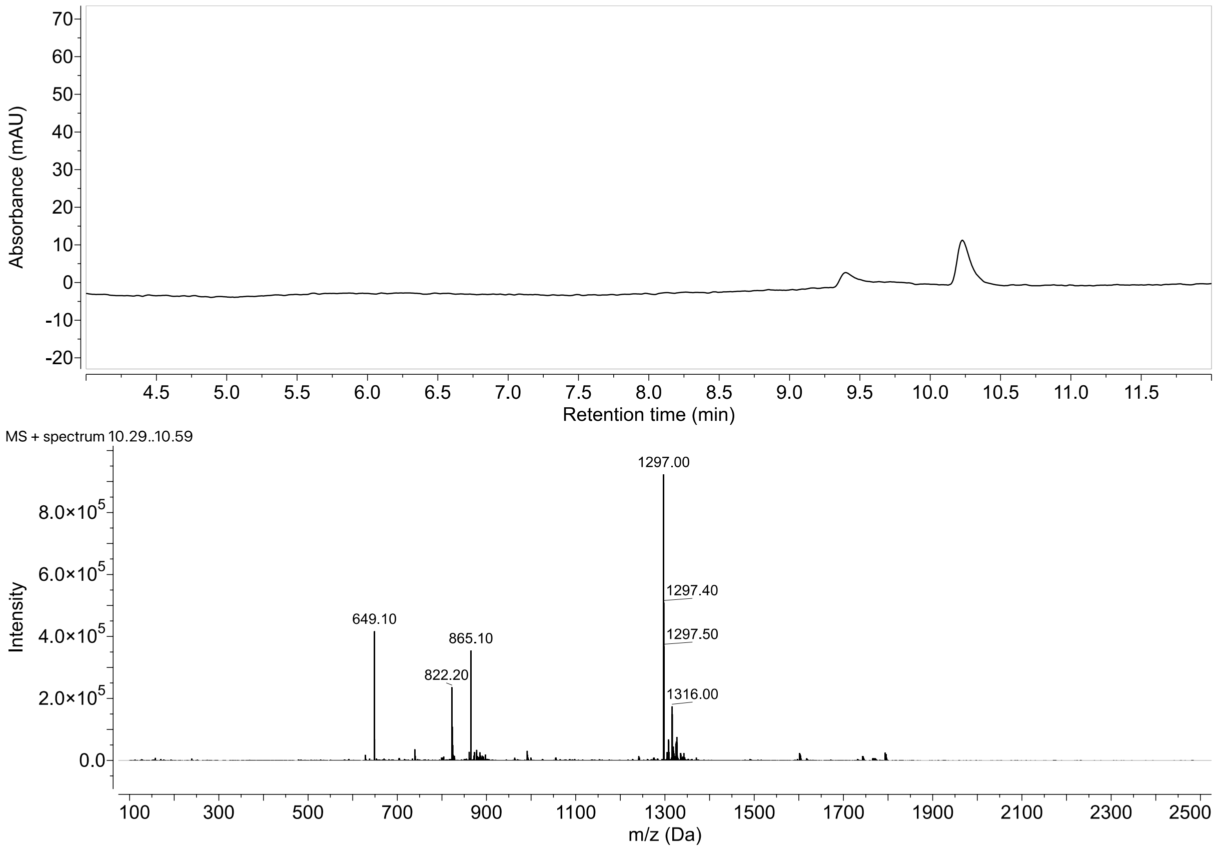

Figure 10. LC (UV absorption at 505nm, desired peak is at 10.24min) and MS Spectra of Compound **(Methac-PEG12-12-9)**.

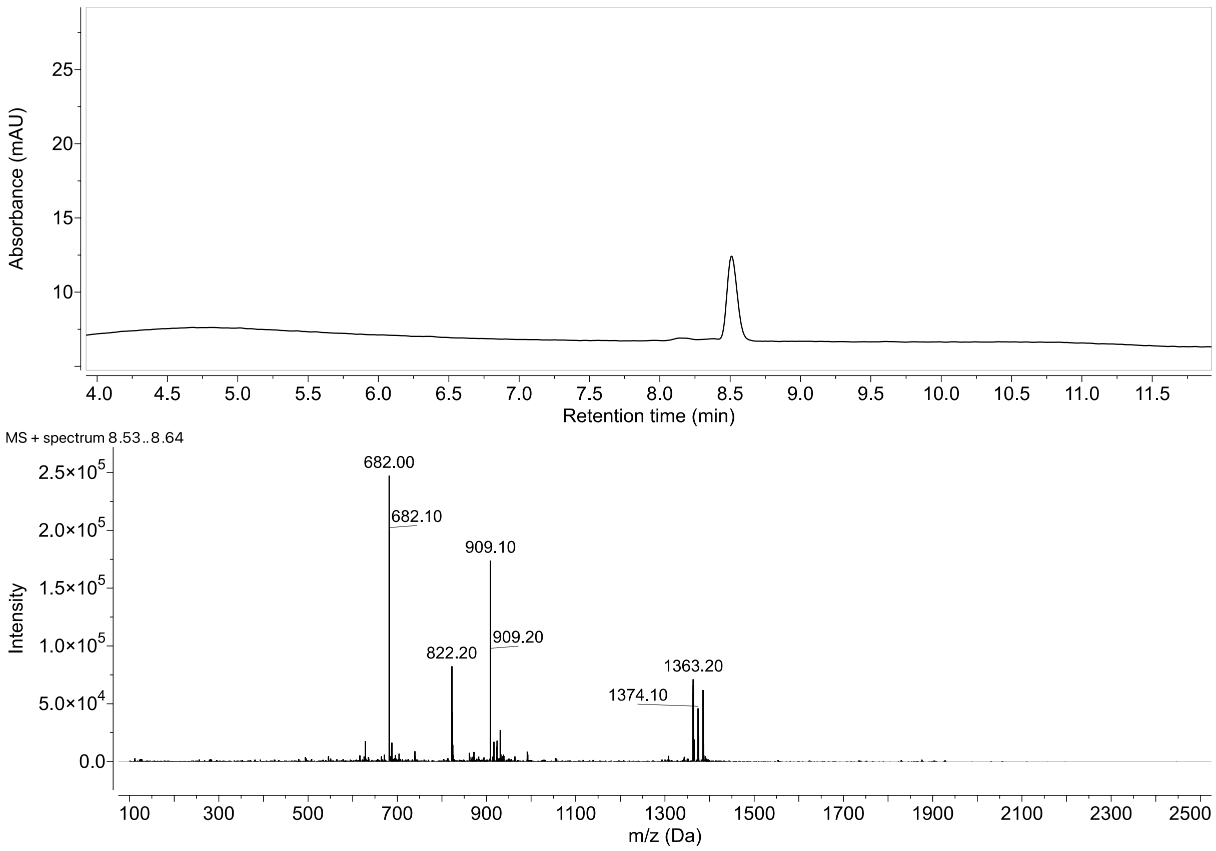

Figure 11. LC (UV absorption at 505nm, desired peak is at 8.51min) and MS Spectra of Compound **(Methac-PEG12-12-10)**.

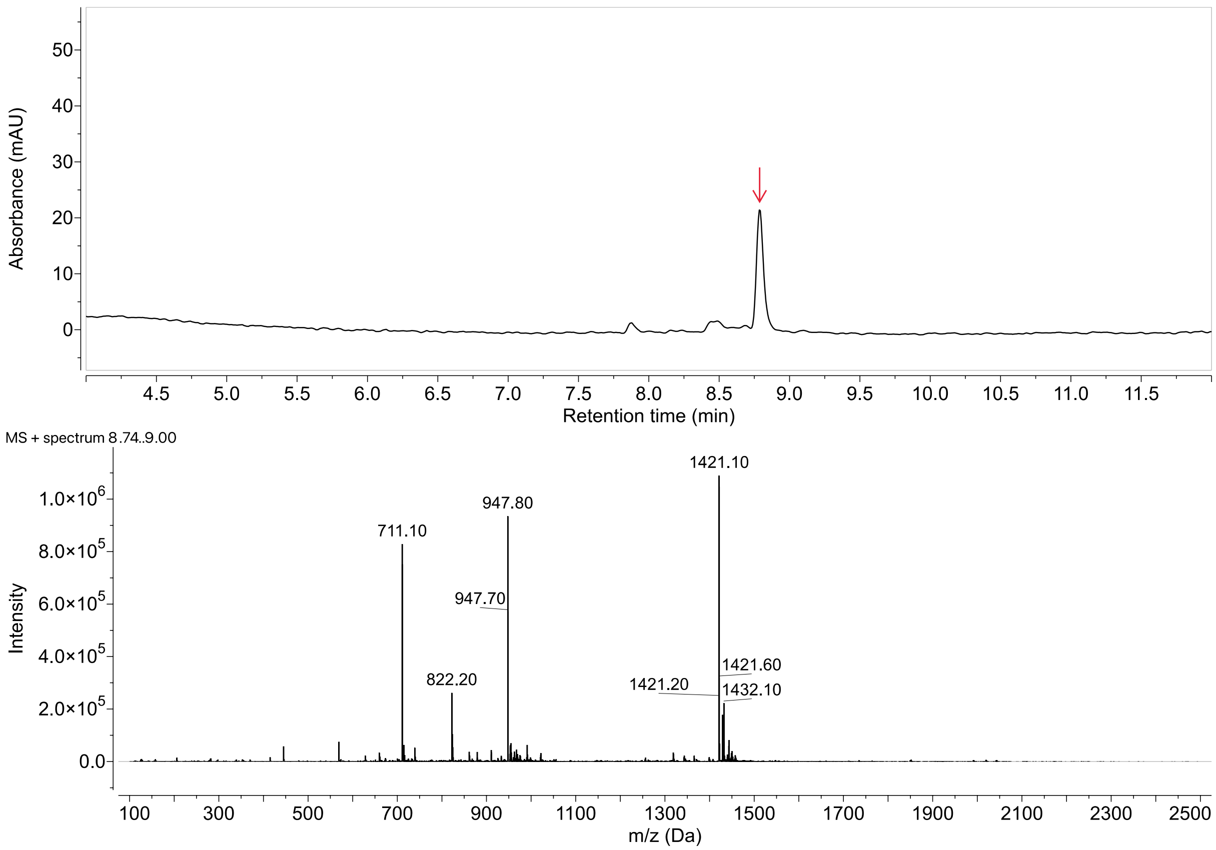

Figure 12. LC (UV absorption at 505nm, desired peak at 8.79min) and MS Spectra of Compound **(Methac-PEG12-12-11)**.

Probe Incorporation Mediated by Enzymes (PRIME)

His$_6$-^{w37v}LplA production in NiCo21 (DE3) *Escherichia coli* (NEB) was as previously described[122]. Briefly, 5 mL precultures in LB containing 100 µg/ml ampicillin were grown overnight at 37°C, 220 rpm, diluted with fresh medium to 500 ml in baffled flasks, and further grown until OD600 reached 0.5. The cultures were induced by 0.1 mM IPTG overnight at 24°C, harvested, resuspended in B-PER (Thermo Fisher Scientific) containing Protease inhibitor cocktail and PMSF, and gently agitated for 10 min at 4°C for cell lysis. The lysates were cleared by centrifugation at 10,000g for 5 min at 4°C. The recombinant His6-W37Vlpla protein was purified using immobilized metal affinity chromatography. Supernatant diluted into 1× Ni-NTA binding buffer was bound to equilibrated Ni-NTA resin for 20 min at 4°C. The resin was added to a spin column, washed thoroughly, and incubated with the Ni-NTA elution buffer for 20 min at 4°C. Recombinant His$_6$-

^{w37v}LplA proteins were then eluted in 50 mM Tris, 300 mM NaCl, 100 mM imidazole, pH 7.8, aliquoted and stored at -80°C.

Picolyl azide (pAz) was synthesized following Uttamapinant et al[122] through sequential steps of Methyl 6-(hydroxymethyl)nicotinate 2, Methyl 6-(hydroxymethyl)nicotinate 3 and 6-azidomethylnicotinic acid 4 syntheses. *Methyl 6-(hydroxymethyl)nicotinate 2 synthesis:* dimethyl 2,5-pyridine dicarboxylate (1.2 mmol)) and anhydrous $CaCl_2$ (205.4 mmol) mixed with anhydrous THF (100 mL) and anhydrous methanol (200 mL) in ice-cold water bath were added NaBH4 (103.1 mmol) and stirred at 0°C for 2 h. Methyl 6-(hydroxymethyl)nicotinate 2 was obtained by quenching the reaction mixture with ice-cold water, extracted four times with chloroform, washed with water, dried with sodium sulfate and concentrated using rotary evaporation.

Methyl 6-(azidomethyl)nicotinate 3 synthesis: anhydrous triethylamine (95.7 mmol) and p-toluenesulfonyl chloride (35.9 mmol) were added to methyl 6-(hydroxymethyl)nicotinate 2 (23.9 mmol) dissolved in anhydrous dichloromethane (240 mL) and stirred at RT for 3 h. Dichloromethane was completely removed using rotary evaporation. Sodium azide (230.9 mmo) was added to the resulting residue dissolved in THF (240 mL), stirred at RT for 24 h, concentrated by rotary evaporation and subsequently diluted in ethyl acetate and water before extracting the three times with ethylacetate, washed with brine solution, dried over Na_2SO_4 and concentrated using rotary evaporation. Methyl 6-(azidomethyl)nicotinate 3 was purified by flash chromatography on silica, using isocratic 4:1 hexanes:ethyl acetate.

6-azidomethylnicotinic acid 4 synthesis: Methyl 6-(azidomethyl)nicotinate 3 (1.32 g) dissolved methanol (68 mL) were hydrolyzed in 1M LiOH in water (20.5 mmo), stirred at RT for 25 min before adding acetic acid (700 µl) and concentrated using rotary evaporation. 6-azidomethylnicotinic acid 4 was purified by flash chromatography on silica, using isocratic ethyl acetate + 1% (v/v) acetic acid. *pAz synthesis*: anhydrous triethylamine (2.53 mmol) and N,N′-disuccinimidyl carbonate (2.53 mmol) were added to 6-azidomethylnicotinic acid 4 (1.68 mmol) dissolved in anhydrous DMF (5 mL) and stirred at RT for 3 h, before dilution with chloroform and water, further extracted three times with chloroform, and washed with brine solution, dried over Na_2SO_4 and concentrated using rotary evaporation. Succinimidyl ester of 6-azidomethylnicotinic acid was purified by flash chromatography on silica, using isocratic 1:1 hexanes:ethyl acetate. Anhydrous triethylamine (1.08 mmol) and 5-aminovaleric acid (1.08 mmol) were added to succinimidyl ester of 6-azidomethylnicotinic acid (0.36 mmol) dissolved in anhydrous DMF (1.75 mL) and stirred at RT for 3 h before concentration using rotary evaporation. pAz was purified by HPLC as above.

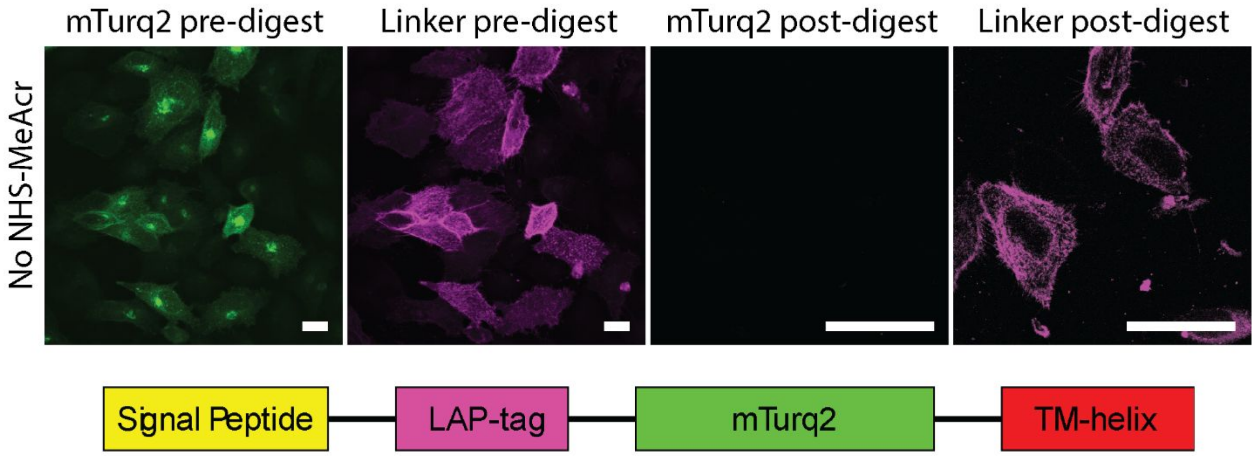

Supplementary Figure 1. Probing small peptide tags with the trifunctional linkers. The 13 amino acid LplA acceptor peptide (LAP) tag was fused to an extracellular leaflet plasma membrane targeting signal peptide, the fluorescent protein mTurquiose2 (mTurq2) and a synthetic transmembrane domain (TM-helix). HeLa cells transfected with the display peptide were first derivatized with a picolyl-azide reagent via LplA enzymatic incorporation, then labeled with the trifunctional linker alkyne-rhodamine-methacrylamide (ARM) via copper-catalyzed alkyne-azide cycloaddition (CuAAC). Samples were embedded into the expansion gel without the general protein anchoring reagent methacrylic acid *N*-hydroxysuccinimide ester (NHSMeAcr). Gelled samples were imaged before and after digestion to demonstrate the ability of the ARM linker for retaining the LAP tag, in contrast to the loss of mTurq2. Scale bars are 20 µm (pre-digest) and 5 µm (post-digest, adjusted for a slight 1.65 expansion in digestion buffer)

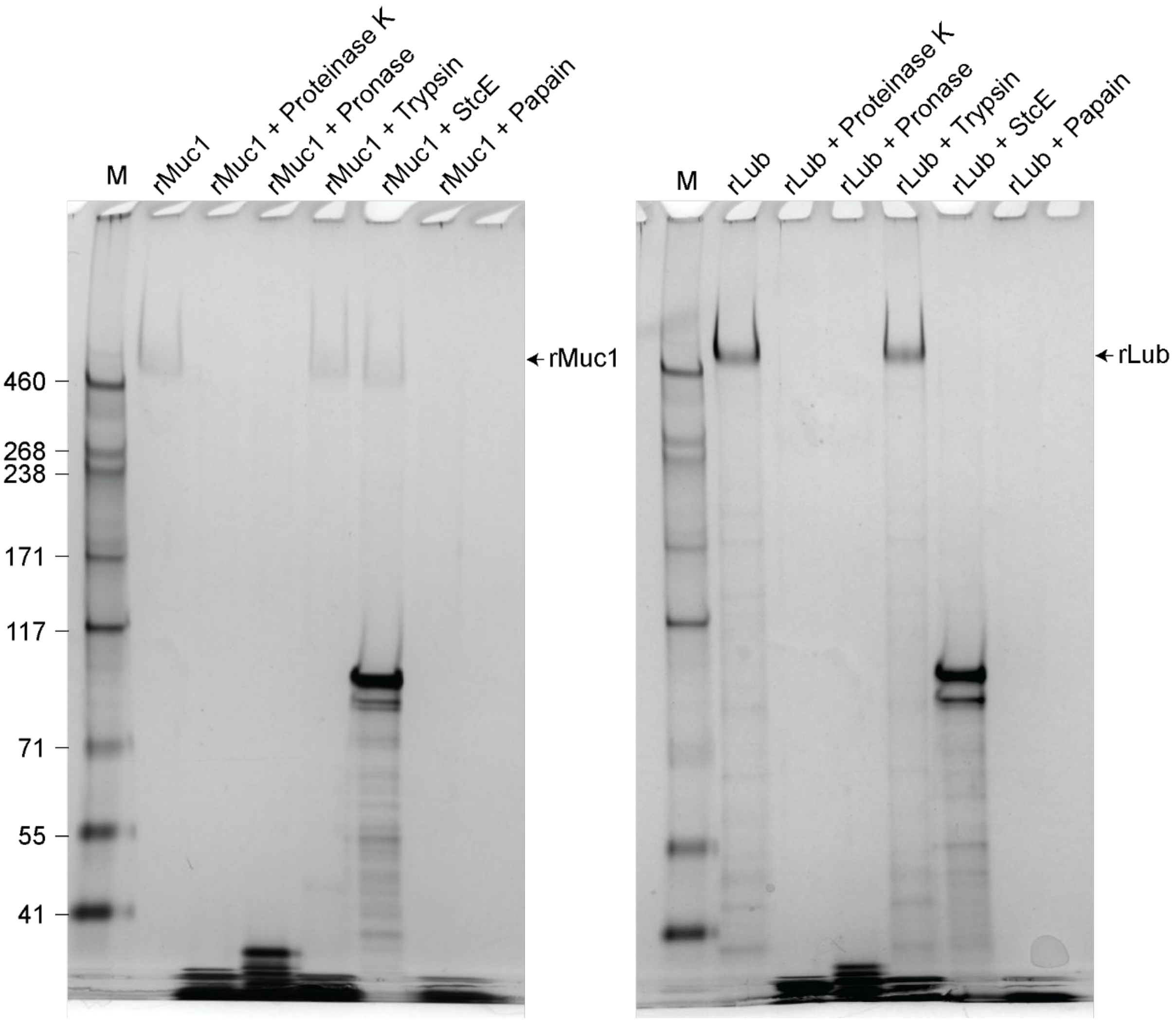

Supplementary Figure 2. Enzymatic digestion of Mucin 1 (left panel) and lubricin (right panel) analyzed by silver-stained SDS-PAGE gels. Lane 1 (M) is the HiMark Pre-stained Protein Standard (Invitrogen). Lane 2 is the recombinant Mucin 1 (rMuc1) or lubricin (rLub). Lanes 3 to 7 are the reaction mixtures of recombinant Muc1 or lubricin with proteinase K, pronase, trypsin, StcE mucinase, or papain, respectively.

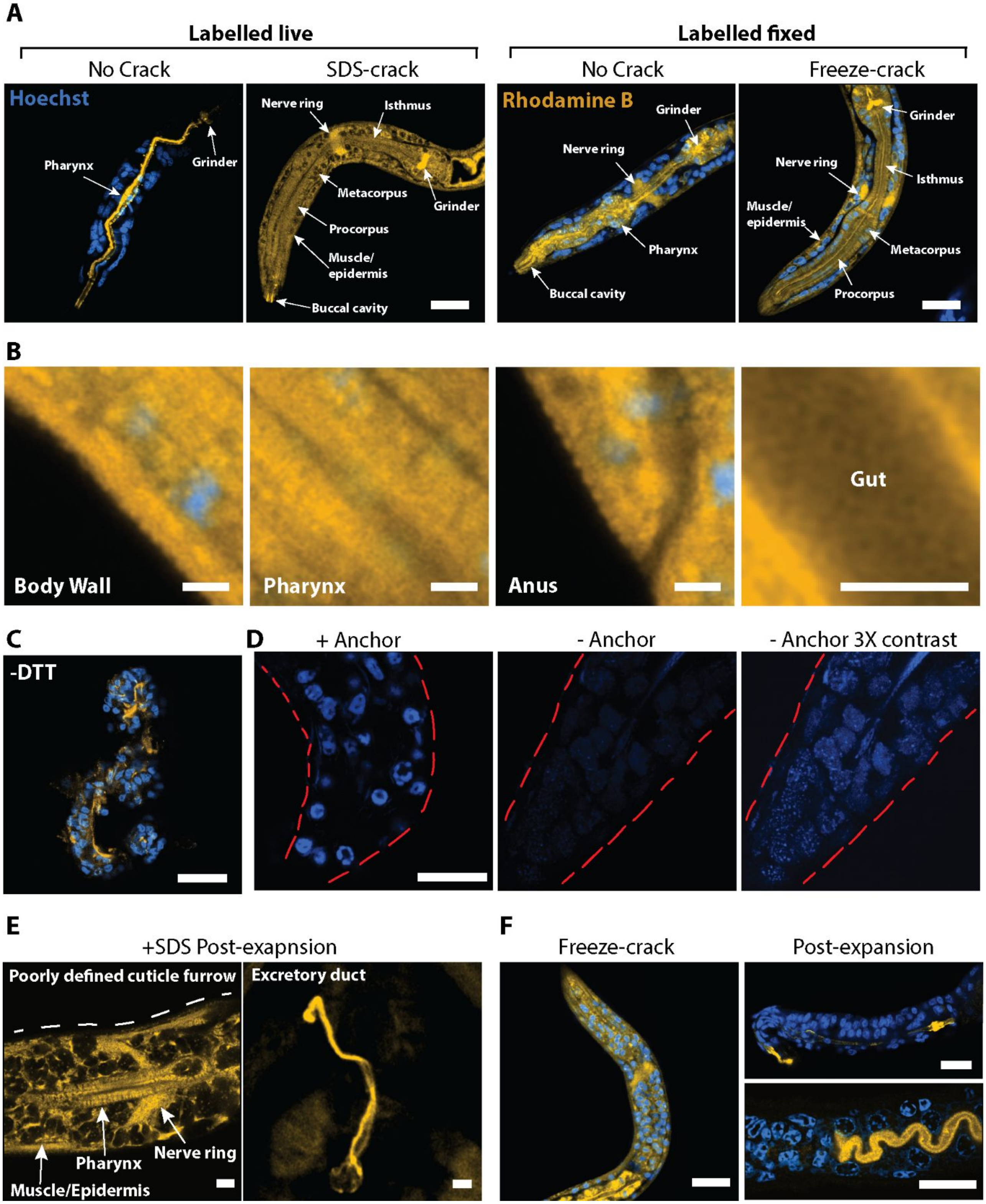
A
Labelled live
Labelled fixed
No Crack
SDS-crack
No Crack
Freeze-crack
Hoechst
Pharynx
Grinder
Nerve ring
Isthmus
Metacorpus
Grinder
Procorpus
Muscle/ epidermis
Buccal cavity
Rhodamine B
Grinder
Nerve ring
Pharynx
Buccal cavity
Grinder
Nerve ring
Isthmus
Muscle/ epidermis
Metacorpus
Procorpus
B
Body Wall
Pharynx
Anus
Gut
C
-DTT
D
+ Anchor
- Anchor
- Anchor 3X contrast
E
+SDS Post-exapnsion
Poorly defined cuticle furrow
Pharynx
Nerve ring
Muscle/Epidermis
Excretory duct
F
Freeze-crack
Post-expansion

Supplementary Figure 3. Protocol optimization for bio-orthogonal labelling of *C. elegans* larvae and expansion microscopy. (A) Cuticle permeabilization by either 0.25% sodium dodecyl sulfate (SDS, SDS-crack) or cycles of freeze-thaw (freeze-crack) enhanced tissue penetration of the DBCO-Rhodamine-Methacrylamide (DRM) linker throughout the entire animal for labelling tetra-acylated azidoacetylgalactosamine (GalNAz). Compared to live larvae, fixed animals allowed better linker penetration without cuticle permeabilization (No Crack). (B) Ultrastructures corresponding to Figures 4C and 5C, such as body wall cuticle furrows and gut microvilli, remained unresolved without the expansion microscopy (ExM) protocol in GalNAz L1 larvae labelled with the DRM linker. (C) Observable structural distortion of expanded samples in the absence of a reducing agent, DTT, which was required for disrupting the cuticle collagen following digestion and gel expansion. (D) DNA retention in ExM was enhanced by the application of methacrylic acid *N*-hydroxysuccinimidyl ester to broadly anchor proteins into the expansion gel matrix. Red dotted lines outline the animals. (E) Brief treatment (2 min) of larvae with the anionic detergent SDS at 0.25% removed the GalNAz-enrichment of cuticle furrows. (F) Representative images of DBCO-PEG$_4$-Sulforhodamine B labelling of fixed GalNAz-fed L1 larvae after cycles of freeze-thaw to enhance tissue penetration. Left, labelled and unexpanded. Right, Post-expansion with protein-retention ExM (proExM) anchoring using MA-NHS. Yellow, Rhodamine. Blue, nuclear stain Hoeschst. Scale bars in (A) 10 µm; (B) 2 µm; (C) 40 µm (unadjusted for expansion factor); and after adjusting for linear expansion factor 3.72X are (D) 5 µm and (E) left, 1 µm and right, 500 nm; (F) left unexpanded, 10 µm, and right, 5 µm (adjusted for linear expansion factor 3.72X).

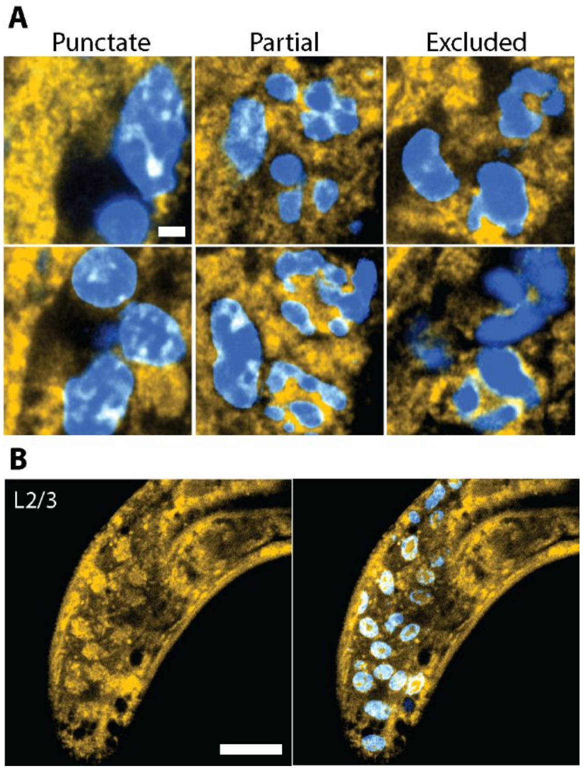

Supplementary Figure 4. Examples of DRM ExM linker labelling cell nuclei. (A) Newly hatched L1 with GalNAz showed various DRM linker labelling patterns in the nucleus of cells around the gut region. Post-expansion, a punctate pattern inside the nucleus (marked by Hoechst) can be observed in some cells. Shown are also examples of partially labelled nucleus regions and other cell types exhibiting a complete lack of DRM linker labelling ("Excluded"). Images shown are mid-plane of z-stacks. (B) Prominent nucleus staining can be seen in older larvae, such as L2/L3 larvae shown. Scale bars: A, 500 nm adjusted for expansion factor of 3.7X and B, 10 μm.
